# General Therapeutics and Materia Medica; Adapted for a Medical Text Book

**Published:** 1850-11

**Authors:** 


ARTICLE III.

*General Therapeutics and Materia Medica; adapted for a Medical Text Book.*---By ROBLEY DUNGLISON, M.D., Professor of Institutes of Medicine, &c., in Jefferson Medical College of Philadelphia; formerly Professor of Materia Medica and Therapeutics in the Universities of Virginia and Maryland, and in Jefferson Medical College of Philadelphia. One hundred and eighty-two illnstrations. Fourth edition, revised and improved. Philadelphia: Lea & Blanchard, 1850; 2 vols., 8vo., pp. 996. (From the Publishers, and for sale by S. C. Griggs & Co., 111 Lake street, Chicago.)

Prof. Dunglison is one of the most voluminous of medical authors, and is in himself almost a refutation of the proverb, "non omnes omnia possumus." That his labors are kindly appreciated may be inferred from the fact that the work before us has already run through three editions, and a fourth has been called for. In this edition the learned and indefatigable author has made extensive additions and alterations, and illustrated it with quite a number of wood-cuts, which add to its value. In our very limited space it is impossible to give anything like an analysis of what we conceive to be its merits or defects as a system of Materia Medica and Therapeutics. As the work has long been before the medical public, and has acquired the reputation of a standard authority, we only deem it necessary to observe that this edition is, in many particulars, an improvement on any that has preceded it. The publishers have, as usual, brought out the work in excellent style.

